# Point of Care Perioperative Coagulation Management in Liver Transplantation and Complete Portal Vein Thrombosis

**DOI:** 10.1155/2014/487364

**Published:** 2014-02-06

**Authors:** Cristiano Piangatelli, Lucia Faloia, Claudia Cristiani, Ilaria Valentini, Marco Vivarelli

**Affiliations:** ^1^Division of Anaesthesia and Resuscitation, Department of Emergency, Ospedali Riuniti, Via Conca 71, 60020 Ancona, Italy; ^2^Department of Emergency, Anesthesia and Resuscitation Unit, Università Politecnica delle Marche, Via Conca 71, 60020 Ancona, Italy; ^3^Department of Liver Surgery and Transplantation, Ospedali Riuniti, Via Conca 71, 60020 Ancona, Italy

## Abstract

Liver transplantation (LT) is a serious hemostatic challenge in patients with portal vein thrombosis (PVT). Advances in monitoring systems have improved surgery in this setting. We report the successful application of a point-of-care (POC) rotational viscoelastic thromboelastometry-guided (TEM) testing system (ROTEM) which allowed management of coagulation during LT in a 64-year-old cirrhotic patient with a model for end-stage liver disease (MELD) score of 16. Perioperatively, the patient showed complete PVT, hepatomegaly, splenomegaly, recanalization of the umbilical vein, and portosystemic shunt. Macroscopic liver and spleen adherences with collateral circulation were evident. Coagulation factors and fibrinolysis were assessed preoperatively and at graft reperfusion to evaluate the need of hemostatic therapy. Based on ROTEM findings, the patient received 16 g of human fibrinogen concentrate, half preoperatively (with prothrombin complex concentrate 2000 IU, tranexamic acid 1 g, and platelets 2 IU), and two doses of 4 g before and after graft reperfusion; we achieved normalization of all monitored parameters. No ischemia-reperfusion syndrome was present. Postoperatively portal vein flux at Color-Doppler ultrasonography was normal. After a 3-day ICU stay, the patient was moved to the Department of Surgery and discharged on day 14. The postoperative course was uneventful and did not require any further haemostatic therapy.

## 1. Introduction 

Chronic liver disease affects hemostasis via three predominant mechanisms: reduced synthesis of coagulation factors and inhibitors, thrombocytopenia and/or thrombocytopathy, and altered fibrinolysis. Liver transplantation (LT) is a serious haemostatic challenge faced by patients with chronic liver disease [1], as the risk of coagulopathic bleeding adds to surgical bleeding. In liver cirrhosis, portal hypertension may induce the formation of collateral vessels that drain portal blood directly into the general circulation, bypassing the liver and causing congestion of the portal area [2]. This situation may dramatically increase surgical bleeding during LT. Portal vein thrombosis (PVT) may decrease the portal flow worsening portal hypertension [3], a condition which shows an incidence of 2–26% in patients with end-stage liver disease which are candidates to LT [4] and who demonstrate exacerbate bleeding during hepatectomy.

LT is always a complex surgical procedure; its complexity increases even more in patients with PVT [5] and in the past PVT used to be considered an absolute contraindication for surgery [6]. However, the advancement in surgical techniques in the last two decades allows now for successfully performing LT in these difficult patients also.

## 2. A Case Report 

We describe a point-of-care (POC) system used to successfully manage intraoperative coagulation in a 64-yearold man with hepatitis C virus- (HCV-) related liver cirrhosis and PVT undergoing LT. Our technique assessed the need of prophylactic intraoperative administration of coagulation factor concentrates based on a thromboelastometry-guided (TEM) viscoelastic testing.

The patient had a model for end-stage liver disease (MELD) score of 16 and was listed for cadaveric liver transplantation. At a perioperative abdominal computer tomography with contrast, the patient showed a complete portal vein thrombosis, hepatomegaly, splenomegaly, recanalization of the umbilical vein, and portosystemic shunt. We obtained normalization of all parameters, respect ROTEM reference values [7] (Table 1). A point-of-care (POC) rotational TEM device (ROTEM, Tem International GmbH, Munich, Germany) was used to perform EXTEM and FIBTEM assays. Coagulation factors and fibrinolysis were performed preoperatively, immediately before graft reperfusion to assess the need of administration of hemostatic therapy, and repeated after 20 min from reperfusion.

The patient received general anesthesia and routine vascular accesses were inserted: the right jugular vein was cannulated with a high-flow trilumen catheter (AVA Edwards Lifesciences, Irvine, CA, USA), and a Swan-Ganz catheter (Edwards Lifesciences, Irvine, CA, USA) was inserted. The right femoral artery was cannulated and a peripheral high-flow catheter was placed in the right arm.

Blood temperature was continuously monitored and maintained at >36°C with forced-air and fluid-warming devices.

After surgical opening of the peritoneum and aspiration of ascites, liver and spleen macroscopic adherences with collateral circulation were evident. The clinical picture was indicative of a potential important surgical bleeding, which could be worsened by a concomitant hepatic insufficiency related bleeding. Perioperatively, ROTEM findings suggested an increased clotting time (CT), a reduction in *α* angle, probably due to a hypofibrinogenemia supported by the value of the maximum clot firmness (MCF) assessed with FIBTEM test, and a modest fibrinolysis, according to the value of EXTEM ML30%. On the basis of these values, which are shown in Table 1, preoperatively the patient received in this order 8 g human fibrinogen concentrate (Haemocomplettan P, CSL Behring, Marburg, Germany), 2,000 IU prothrombin complex concentrate (Human Complex Kedrion Biopharma, Castelvecchio Pascoli, Italy), 2 IU platelet concentrate, and 1 g tranexamic acid.

Immediately after therapy, the TEM tests were repeated and showed normalization of parameters without any evidence of pathological clot lysis (see Table 1). Warm ischaemia time was approximately 35 min, fully consistent with usual practice (approximately 35 min).

ROTEM testing performed before reperfusion of the graft showed low levels of blood fibrinogen leading to the administration of a first 4 g dose of fibrinogen. Measurements were repeated 20 mins after reperfusion and values resulting indicated a reduced strength and/or stability of the clot; thus, a further dose of 4 g fibrinogen concentrate was administered. We obtained normalization of all parameters (Table 1). In total the patient received 8 g of fibrinogen concentrate. No ongoing bleeding was present at the time of fibrinogen administration. Arterial and biliary anastomosis were completed concurrently. No evidence of an ischaemia-reperfusion syndrome—a heparin effect of coagulation disorders after reperfusion—was apparent.

The patient was awakened and extubated as soon as the surgical intervention was concluded, as previously described [8]. After 12 hours from transplant, 100 mg daily ASA was started to reduce the risk of hepatic artery thrombosis. Portal vein flux measured with Color-Doppler ultrasonography during the patient's stay in intensive care unit (ICU) was normal. After a 3-day ICU stay, the patient was moved to the Department of Surgery and discharged on day 14 from surgery. The postoperative course was uneventful and did not require further haemostatic therapy.

## 3. Discussion 

Coagulation disorders in liver cirrhosis are well documented. The hemostatic profile of a patient with chronic liver failure typically includes thrombocytopenia, reduced levels of coagulation factors and inhibitors, and unbalanced fibrinolysis [9]. In addition, LT is a challenging surgical procedure and is often associated with extensive bleeding. This surgery has the highest risk of hemorrhage due to the difficult removal of the native liver consequent to adherences and collateral vessels. Portal thrombosis may worsen portal hypertension, thus increasing bleeding during hepatectomy. The intraoperative bleeding can be even more worsened by a deficit of coagulation factors and lower force on coagulum, which are secondary to low levels of fibrinogen and/or platelet.

Viscoelastic testing enables dynamic assessment of clotting in whole blood and provides information on the procoagulant, anticoagulant, and fibrinolytic pathways, thus being a perioperative invaluable tool in LT [10–14]. The successful use of a POC-based ROTEM-guided algorithm for perioperative coagulation management of liver transplantation was previously described by Goerlinger et al. [14]. In contrast, several studies have demonstrated a poor correlation between alterations in conventional laboratory coagulation tests (PT, INR and aPTT) and any bleeding tendency caused by the rebalancing of procoagulant and anticoagulant factors in chronic liver failure [15].

In this case we adopted a restrictive transfusion strategy, associated with limited intraoperative blood loss, which allowed a correction of the CT with a prothrombin complex with coagulation factors (factors II, VII, IX, and X) that contain, also, natural inhibitors (such as proteins C and S).

In our case, we used a POC ROTEM System to identify and treat haemocoagulative alterations. Lisman and Leebeek [15] demonstrated in cirrhotic patients undergoing surgery that the hemostatic balance was preserved, with extremely low reserve. In fact, our patient showed hypofibrinogenemia, causing a reduced clot strength for limited interaction between fibrinogen and platelets.

Thrombocytopenia is present in cirrhotic patients due to splenic sequestration; as a consequence the action of intraoperative administration of platelets is short; moreover, platelet infusion after the graft reperfusion causes an active translocation of platelets into sinusoidal and Disse's space with intrahepatic microthrombosis [16]. Platelets, moreover, are the blood component with the higher risk to induce TRALI (transfusion-related acute lung injury) due to the presence of many surface antigens [17]. Therefore, the administration of fibrinogen allows an improvement of the strength of the clot, also in the patients with low platelet count as shown by Lang et al. [18].

In summary, in the reported case we have demonstrated that optimizing coagulation through goal-directed, TEM-guided use of coagulation factor concentrates before and during surgery can reduce coagulopathic bleeding and subsequent PRCB and FFP (fresh frozen plasma) transfusion requirements in patients with PVT submitted to LT.

## Figures and Tables

**Table 1:** Preoperative ROTEM parameters before and after haemostatic therapy and perioperative values at graft reperfusion and after two rounds of haemostatic therapy.

ROTEM parameters assessed	Range of normal values	Preoperative values		Peri-operative values at graft reperfusion		
		Before therapy	After therapy*	Before reperfusion	After the 1st 4 g dose of fibrinogen concentrate	After the 2nd 4 g dose of fibrinogen concentrate (20 min from reperfusion)
EXTEM clotting time, CT (s)	42–74	28	54	42	43	43
EXTEM maximum clot firmness, mCF (mm)	49–71	35	65	36	50	61
EXTEM maximum lysis 30 min post CT, ML_30_ (%)	0–18	5	0	0	1	0
EXTEM *α*-angle (°)	63–81	28	70	40	66	72
FIBTEM maximum clot firmness, MCF (mm)	9–25	6	16	6	9	15

*Fibrinogen concentrate 8 g, prothrombin complex concentrate 2000 IU, tranexamic acid 1 g, and platelets 2 IU. EXTEM: extrinsic thromboelastometry assay incorporating recombinant tissue factor as activation enhancer; FIBTEM: fibrinogen thromboelastometry assay based on the EXTEM assay and incorporating cytochalasin D as platelet inhibitor; EXTEM *α*-angle (°): angle between the baseline and target to the clotting curve through the 2 mm point, it represents kinetic of platelet and fibrin polymerization.

## References

[1] Roberts LN, Patel RK, Arya R (2010). Haemostasis and thrombosis in liver disease. *British Journal of Haematology*.

[2] Cichoz-Lach H, Celiński K, Słomka M, Kasztelan-Szczerbińska B (2008). Pathophysiology of portal hypertension. *Journal of Physiology and Pharmacology*.

[3] Zhang D, Hao J, Yang N (2010). Protein C and D-dimer are related to portal vein thrombosis in patients with liver cirrhosis. *Journal of Gastroenterology and Hepatology*.

[4] Bhangui P, Lim C, Salloum C (2011). Caval inflow to the graft for liver transplantation in patients with diffuse portal vein thrombosis: a 12-Year experience. *Annals of Surgery*.

[5] Hoekstra J, Janssen HLA (2009). Vascular liver disorders (II): portal vein thrombosis. *Netherlands Journal of Medicine*.

[6] Tao Y-F, Teng F, Wang Z-X (2009). Liver transplant recipients with portal vein thrombosis: a single center retrospective study. *Hepatobiliary and Pancreatic Diseases International*.

[7] Görlinger K, Jambor C, Hanke AA (2007). Perioperative coagulation management and control of platelet transfusion by point-of-care platelet function analysis. *Transfusion Medicine and Hemotherapy*.

[8] Biancofiore G, Bindi ML, Romanelli AM (2005). Fast track in liver transplantation: 5 Years’ experience. *European Journal of Anaesthesiology*.

[9] Lisman T, Porte RJ (2010). Rebalanced hemostasis in patients with liver disease: evidence and clinical consequences. *Blood*.

[10] Trzebicki J, Flakiewicz E, Kosieradzki M (2010). The use of thromboelastometry in the assessment of hemostasis during orthotopic liver transplantation reduces the demand for blood products. *Annals of Transplantation*.

[11] Wang S-C, Shieh J-F, Chang K-Y (2010). Thromboelastography-guided transfusion decreases intraoperative blood transfusion during orthotopic liver transplantation: randomized clinical trial. *Transplantation Proceedings*.

[12] Coakley M, Reddy K, Mackie I, Mallett S (2006). Transfusion triggers in orthotopic liver transplantation: a comparison of the thromboelastometry analyzer, the thromboelastogram, and conventional coagulation tests. *Journal of Cardiothoracic and Vascular Anesthesia*.

[13] Stancheva A, Spassov L, Tzatchev K (2011). Correlation between rotation thrombelastometry ROTEM analysis and standard haemostatic parameters during liver transplantation. *Clinical Laboratory*.

[14] Goerlinger K, Dirkmann D, Hanke A, Dusse F, Hartmann M (2008). ROTEM-based algorithm for point-of-care coagulation management in visceral surgery and liver transplantation: experience of eight years and 829 LTX. *Liver Transpantation*.

[15] Lisman T, Leebeek FWG (2007). Hemostatic alterations in liver disease: a review on pathophysiology, clinical consequences, and treatment. *Digestive Surgery*.

[16] Sindram D, Porte RJ, Hoffman MR, Bentley RC, Clavien PA (2001). Synergism between platelets and leukocytes in inducing endothelial cell apoptosis in the cold ischemic rat liver: a Kupffer cell-mediated injury. *The FASEB Journal*.

[17] Liumbruno G, Bennardello F, Lattanzio A, Piccoli P, Rossetti G (2009). Recommendations for the transfusion of plasma and platelets. *Blood Transfusion*.

[18] Lang T, Johanning K, Metzler H (2009). The effects of fibrinogen levels on thromboelastometric variables in the presence of thrombocytopenia. *Anesthesia and Analgesia*.

